# Structural and molecular myelination deficits occur prior to neuronal loss in the YAC128 and BACHD models of Huntington disease

**DOI:** 10.1093/hmg/ddw122

**Published:** 2016-04-28

**Authors:** Roy Tang Yi Teo, Xin Hong, Libo Yu-Taeger, Yihui Huang, Liang Juin Tan, Yuanyun Xie, Xuan Vinh To, Ling Guo, Reshmi Rajendran, Arianna Novati, Carsten Calaminus, Olaf Riess, Michael R. Hayden, Huu P. Nguyen, Kai-Hsiang Chuang, Mahmoud A. Pouladi

**Affiliations:** 1 Translational Laboratory in Genetic Medicine, Agency for Science, Technology and Research, Singapore (A*STAR), Singapore 138648, Singapore; 2 Singapore Bioimaging Consortium, Agency for Science, Technology and Research, Singapore 138648, Singapore; 3 Institute of Medical Genetics and Applied Genomics, University of Tuebingen, 72076 Tuebingen, Germany; 4 Centre for Rare Diseases, University of Tuebingen, 72076 Tuebingen, Germany; 5 Centre for Molecular Medicine and Therapeutics, Child and Family Research Institute, University of British Columbia, Vancouver, BC V5Z 4H4, Canada; 6 Werner Siemens Imaging Center, Department of Preclinical Imaging and Radiopharmacy, University of Tuebingen, 72076 Tuebingen, Germany; 7 Department of Medicine, Yong Loo Lin School of Medicine, National University of Singapore, Singapore 117597, Singapore

## Abstract

White matter (WM) atrophy is a significant feature of Huntington disease (HD), although its aetiology and early pathological manifestations remain poorly defined. In this study, we aimed to characterize WM-related features in the transgenic YAC128 and BACHD models of HD. Using diffusion tensor magnetic resonance imaging (DT-MRI), we demonstrate that microstructural WM abnormalities occur from an early age in YAC128 mice. Similarly, electron microscopy analysis of myelinated fibres of the corpus callosum indicated that myelin sheaths are thinner in YAC128 mice as early as 1.5 months of age, well before any neuronal loss can be detected. Transcript levels of myelin-related genes in striatal and cortical tissues were significantly lower in YAC128 mice from 2 weeks of age, and these findings were replicated in differentiated primary oligodendrocytes from YAC128 mice, suggesting a possible mechanistic explanation for the observed structural deficits. Concordant with these observations, we demonstrate reduced expression of myelin-related genes at 3 months of age and WM microstructural abnormalities using DT-MRI at 12 months of age in the BACHD rats. These findings indicate that WM deficits in HD are an early phenotype associated with cell-intrinsic effects of mutant huntingtin on myelin-related transcripts in oligodendrocytes, and raise the possibility that WM abnormalities may be an early contributing factor to the pathogenesis of HD.

## Introduction

Huntington disease (HD) is an autosomal dominant hereditary disorder characterized by loss of motor control, cognitive deficits and psychiatric disturbances (1). Although preferential degeneration of medium spiny neurons in the caudate and putamen regions of the basal ganglia has long been considered the major neuropathological hallmark of HD, myelin breakdown and white matter (WM) atrophy also appear to be universal features of the disease (2–5). Indeed, in post-mortem brain tissue of HD patients, there is marked loss of myelin and WM volume, as well as significant changes in the numbers and turnover rate of oligodendrocytes, the myelinating cells of the central nervous system (6–8). Transcriptional analyses have further shown that the levels of a large number of myelin-related transcripts are altered in HD brain tissues (9). These findings clearly implicate WM atrophy in the pathology of HD.

A long-held assumption regarding the aetiology of WM atrophy in HD is that it is simply a secondary outcome of the progressive neuronal loss that manifests with advancing disease. However, imaging-based measures that allow the non-invasive assessment of brain structure and function during the early stages of disease have demonstrated that WM abnormalities are in fact an early event. Indeed, structural magnetic resonance imaging (MRI) studies of pre-manifest HD gene carriers reveal that progressive loss of WM volume can be observed many years before disease onset (3,10,11). In addition to structural MRI, magnetic resonance diffusion tensor imaging (MR-DTI), which has been widely used to examine the integrity of neuronal tract connectivity, suggests the presence of microstructural abnormalities in myelinated tracts not only in patients with HD but also in pre-symptomatic gene carriers (12–14). While these studies support the occurrence of WM abnormalities as an early event in HD, they do not preclude the possibility that these changes are secondary to neuronal loss. Furthermore, the nature of these WM abnormalities at a molecular and microstructural level remains poorly defined.

YAC128 HD mice carry a transgene that expresses the full length human huntingtin (HTT) protein with 128 polyglutamine repeats, and develop a number of progressive motor (15,16), cognitive (17) and affective phenotypes (18,19) that mimic the symptoms observed in patients with HD. YAC128 HD mice also develop neuropathological and molecular phenotypes that mimic key aspects of brain pathology in human HD, including transcriptional dysregulation as early as 3 months of age (20–22) and preferential striatal neuronal loss starting at 12 months of age (15).

To better characterize the nature of WM changes in HD and determine their importance in HD pathogenesis, we analyse changes in WM structure and myelin-related gene expression in the YAC128 mouse model. We examine the microstructure of the WM-rich regions of the brain using longitudinal MR-DTI, and the thickness of the myelin sheaths of YAC128 mice by electron microscopy (EM), from 1.5 months of age prior to the detection of any neuronal loss. We also analyse the levels of myelin-related transcripts in striatal and cortical tissues in YAC128 HD mice from 2 weeks of age to determine the effects of mutant HTT on early myelination, and examine whether these changes are intrinsic to oligodendrocytes using differentiated primary oligodendrocyte cells. Finally, to corroborate our findings, we evaluate the MR-DTI phenotypes and the expression of myelin-related genes in the transgenic BACHD rat model of HD (23). Our results are important for understanding the contribution of WM abnormalities to the pathogenesis of HD.

## Results

### MR-DTI reveals early microstructural WM abnormalities in YAC128 HD mice

**Figure 1.**
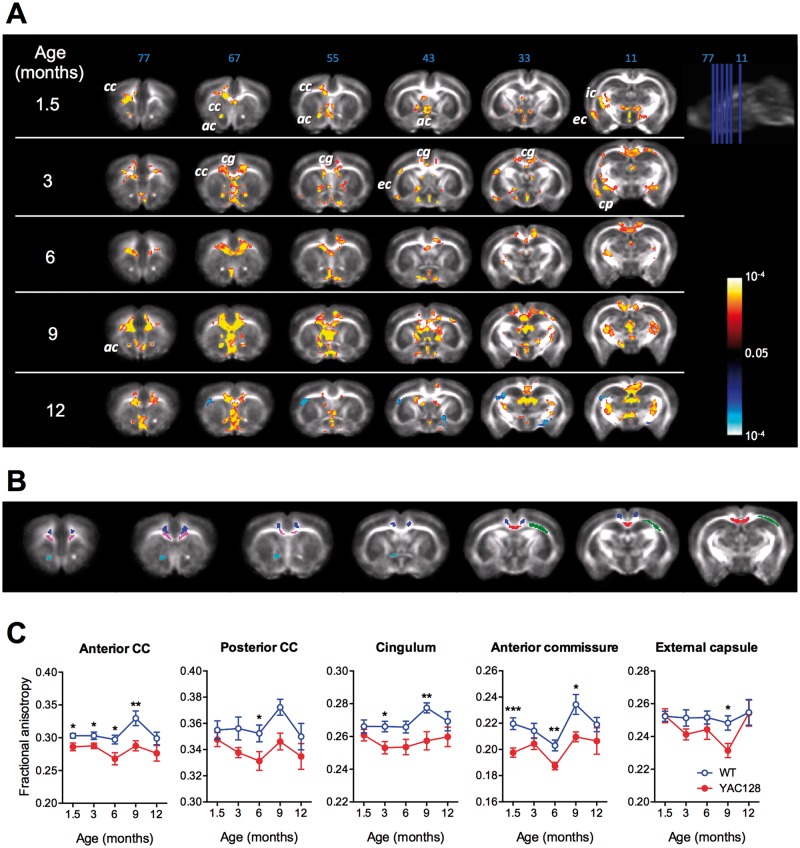
Longitudinal magnetic resonance DTI reveals abnormal white matter microstructure in YAC128 HD mice. (**A**) Voxel-wise comparison of FA values between YAC128 and WT at each time point (each row) at different slice positions (columns). Yellow-red indicates WT > YAC128, blue-light blue indicates YAC128 > WT. ac, anterior commissure; cc, corpus callosum; cg, cingulum; cp, cerebral peduncle; ec, external capsule; ic, internal capsule. (**B**) Region of interest analysis of FA. Examples of ROIs drawn on the FA time-point template. Magenta, anterior corpus callosum; red, posterior corpus callosum; light blue, anterior commissure; dark blue, cingulum; green, external capsule. (**C**) Comparisons of mean FA in each ROI at the indicated time points between YAC128 and WT mice. *n* = 8 (4 males) for WT mice; 8 (4 males) for YAC128 mice; * *P* < 0.05; ** *P* < 0.01.

DTI is a non-invasive approach widely used to assess the integrity of WM microstructure. The principal metric derived from MR-DTI is fractional anisotropy (FA), a measure of the directionality of water diffusion. In fibre bundles with coherent orientation, higher FA values indicate that diffusion occurs primarily along the fibre orientation, correlating with higher overall integrity of the WM microstructure and organization. Voxel-wise analysis indicated that FA values were significantly lower in the WM-rich brain regions of YAC128 mice compared with wild-type (WT) mice, including in the anterior commissure (AC), corpus callosum (CC), internal capsule and external capsule (EC) from 1.5 months of age, and in the cingulum (CG) and cerebral peduncle from 3 months of age (Fig. 1A and B; Supplementary Material, Table S1). Quantification of the FA values in the different brain regions indicated significant reductions over the first year of life in YAC128 mice compared with WT littermates (Fig 1C). These MR-DTI results indicate that WM microstructure abnormalities are present in YAC128 HD mice early on, before the manifestation of behavioural deficits and neuronal atrophy.

**Table 1.** Primers used for cDNA analyses

Target	Forward primer (5′–3′)	Reverse primer (5′–3′)
Mouse MOG	AGCTGCTTCCTCTCCCTTCTC	ACTAAAGCCCGGATGGGATAC
Mouse MBP	GGCCAGTAAGGATGGAGAGAT	CCTCTGAGGCCGTCTGAGA
Mouse CNP	TTTACCCGCAAAAGCCACACA	CACCGTGTCCTCATCTTGAAG
Mouse beta-actin	GGCTGTATTCCCCTCCATCG	CCAGTTGGTAACAATGCCATGT
Rat MOG	AACTCCGTGCAGAAGTCGAG	AACTGTCCTGCCAGTCTTCG
Rat MBP	AGGACCCAAGATGAAAACCCAG	GATGGAGGGGGTGTACGA
Rat CNP	ATGCCCAACAGGATGTGGTG	AGAGGGCAGAGATGGACAGT
Rat ATP5B	GGGTACAATGCAGGAAAGAATC	GGGTACAATGCAGGAAAGAATC
Rat Eif4a2	AAATGCATGCCAGGGACTTCACAG	TTGTTGCACATCAATCCCACGAGC
Rat CanX3	TGTCTGGCAGCGACCTATGATTGA	TCCTTGGTTTCCAGATTCCCTGGT

### Ultrastructural quantitative analysis of myelination in YAC128 HD mice

**Figure 2.**
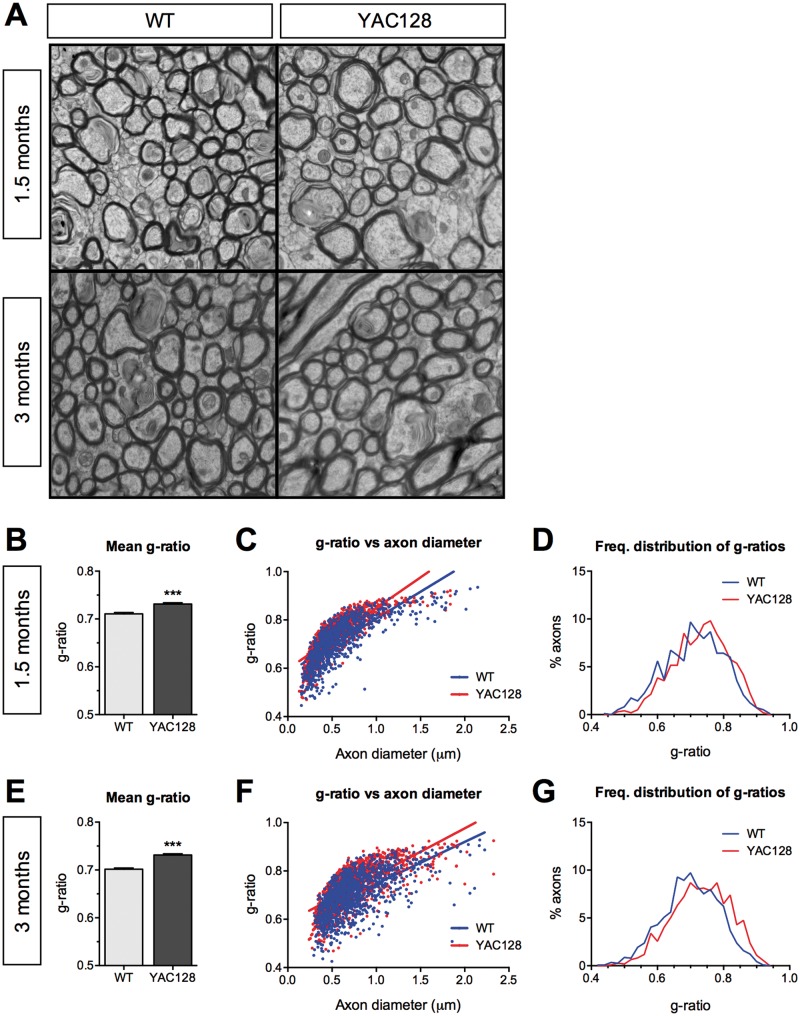
Electronic microscopy analysis shows thinner myelin sheaths in YAC128 HD mice. (**A**) Representative EM images taken from the corpus callosum of WT and YAC128 mice at 1.5 and 3 months of age. (**B**) Mean g-ratios were higher in YAC128 mice compared with their WT littermates, indicating that YAC128 mice had thinner myelin sheaths at 1.5 months of age. (**C**) Scatter plot of g-ratios against axonal diameters with linear regression. The slope of the best-fit line was significantly higher for YAC128 compared with WT mice (*P* = 0.0004), indicating thinner myelin sheaths in YAC128 HD mice at 1.5 months of age. (**D**) Frequency distribution of g-ratios of round healthy axons in YAC128 mice at 1.5 months are shifted to the right, suggesting larger numbers of axons with thinner myelin sheaths. (**E**–**G**) The mean g-ratios, linear regression of g-ratios and axonal diameter, and frequency distribution of g-ratios in WT and YAC128 HD mice at 3 months showed the same trends as seen at 1.5 months of age. (B and E) Error bars represent the standard error of the mean. (B–G) *n* = 3 mice (male) per genotype; ∼500 axons analysed per mouse. *** *P* < 0.001.

To examine whether the observed FA deficits are associated with altered myelination, we used EM on an independent cohort of mice to visualize myelinated fibres in the CC at 1.5 and 3 months of age (Fig. 2A). G-ratios, a measure of myelin sheath thickness calculated as the ratio of axon diameter to myelinated fibre diameter, were determined for YAC128 HD mice and WT littermates. The mean g-ratio of myelinated axons from YAC128 HD mice was significantly higher for YAC128 HD mice compared with WT controls at both 1.5 and 3 months of age, indicating that the myelin sheaths were thinner in YAC128 HD mice (Fig. 2B and E). Plotting g-ratios against axonal diameters demonstrated that g-ratios of larger calibre axons were higher in YAC128 HD mice compared with WT littermates (Fig. 2C and F). In line with these findings, frequency distributions of g-ratios demonstrated a shift to the right in the population, suggesting a moderate thinning of myelin sheaths in YAC128 HD mice (Fig. 2D and G). These changes are observed before the manifestation of behavioural disease phenotypes in 1.5-month-old mice, and persist following onset of phenotypes in 3-month-old mice.

### RNA transcripts of myelin-related genes are altered in pre-manifest YAC128 HD mice

**Figure 3.**
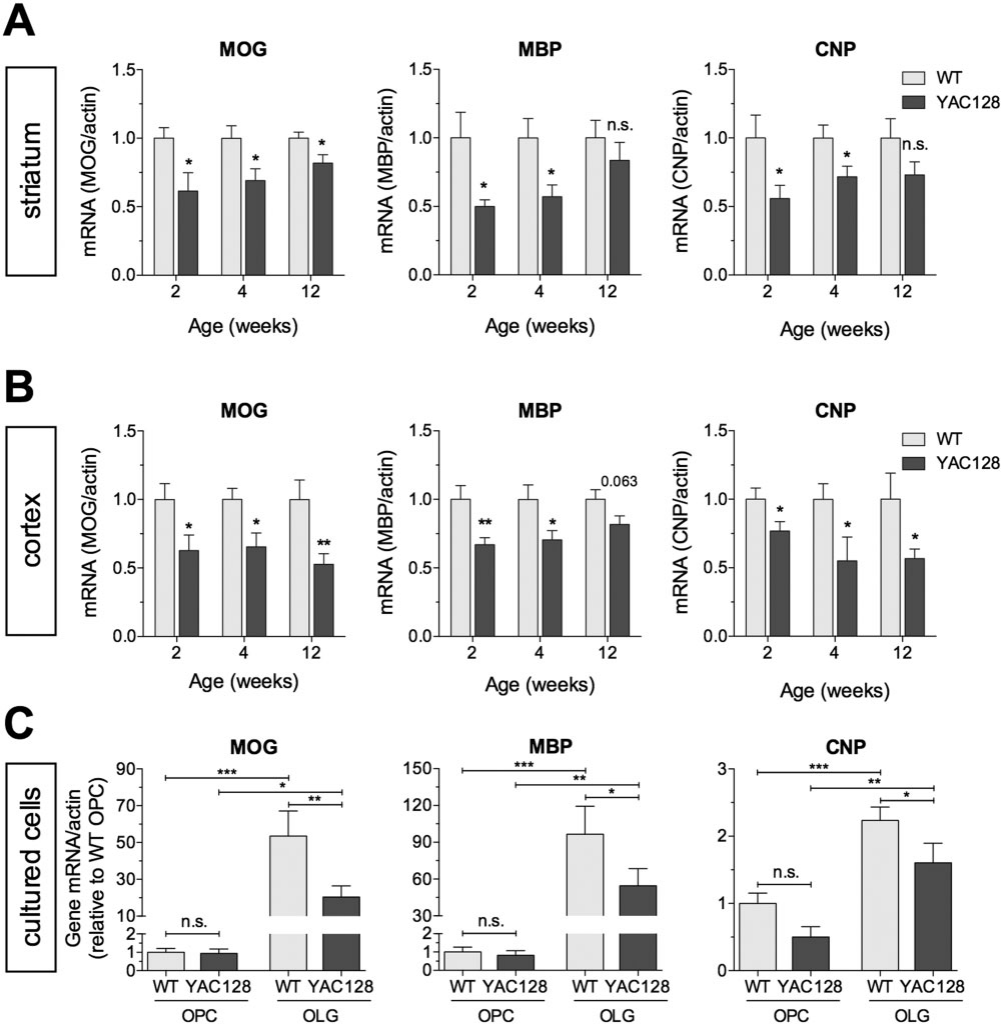
Reduced expression of myelin-related genes in YAC128 HD mice. Lower levels of mRNA transcript of MOG, MBP and CNP in the striatum (**A**) and cortex (**B**) of YAC128 HD mice at 2, 4 and 12 weeks of age compared with WT littermates (*n* = 6–10/genotype/time-point male mice). (**C**) mRNA transcript levels of MOG, MBP and CNP in OPCs immediately after isolation from P6–P7 YAC128 and WT littermate pups and after 7 days in oligodendrocyte differentiation media (*n* = 6–10). No significant differences were observed for any of the investigated transcripts in OPCs. After 7 days in oligodendrocyte differentiation media, a significant upregulation of MOG, MBP and CNP transcripts occurred in both WT and YAC128 mice, but the levels of transcripts for all three genes are significantly lower in YAC128 mice compared with those of WT littermates. All values were normalized to their respective beta-actin levels. (A–C) All values were normalized to their respective beta-actin levels. OPC, oligodendrocyte precursor cell; OLG, oligodendrocyte. * *P* < 0.05; ** *P* < 0.01; *** *P* < 0.001; n.s. = not significant.

To investigate whether changes in myelin-related gene expression are present early in the course of disease prior to neuronal loss, we measured the levels of the pan-oligodendrocyte marker 2′,3′-cyclic-nucleotide 3′-phosphodiesterase (CNP), and the mature myelinating oligodendrocyte markers myelin basic protein (MBP), and myelin oligodendrocyte glycoprotein (MOG) in the striatal and cortical tissues of YAC128 mice and littermate controls at 2, 4 and 12 weeks of age. Compared with WT mice, YAC128 mice had significantly lower levels of CNP (56–72%), MBP (50–57%) and MOG (61–69%) at 2 and 4 weeks of age, and reduced levels of MOG were still apparent at 12 weeks (Fig. 3A). In the cortex, YAC128 mice also had significantly lower levels of CNP (55–77%) and MOG (63–65%) at all time points and significantly lower levels of MBP (67–70%) at 2 and 4 weeks of age (Fig. 3B). This suggests that the thinner myelin sheaths observed in YAC128 HD mice may partly be a consequence of deficits in myelin protein constituents.

### Expression of myelin-related genes in primary differentiated oligodendrocytes

To determine whether changes in myelin-related gene expression reflect a cell-intrinsic effect of mutant HTT in oligodendrocytes, we isolated oligodendrocyte precursor cells (OPCs) from P7 pups using magnetic-activated cell sorting, and cultured them in oligodendrocyte differentiation media over a 7-day period. Expression levels of MOG, MBP and CNP were similar in OPCs from YAC128 and WT mice directly *ex vivo* (Fig. 3C), and a significant induction in transcript levels was observed in both YAC128 and WT mice following the 7-day differentiation period (Fig. 3C). However, the expression levels of MOG, MBP and CNP in differentiated oligodendrocytes were significantly lower in YAC128 compared with WT mice (Fig. 3C). These findings are consistent with the reduced expression of myelin-related gene transcripts in the striatal and cortical tissues of YAC128 mice (Fig. 3A and B), and suggest that the observed deficits reflect cell-intrinsic effects of mutant HTT in oligodendroglial cells.

### Reduced expression of myelin-related genes and abnormal WM microstructure in BACHD rats

**Figure 4.**
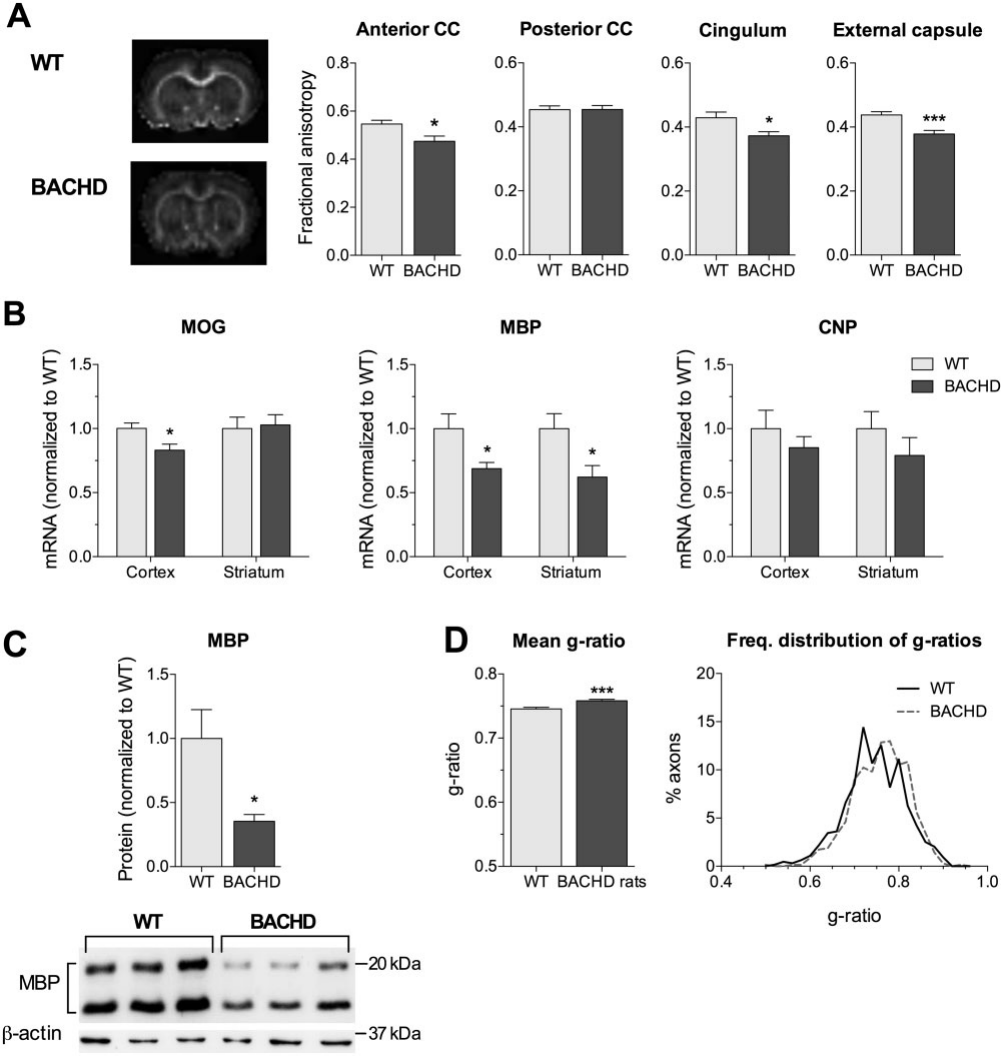
Abnormal white matter microstructure and reduced myelin-related gene expression in BACHD rats. (**A**) Reduced FA values in white matter-rich regions in BACHD rats compared with WT littermates at 12 months of age. cc, corpus callosum. (**B**) Lower levels of mRNA transcripts of myelin-related genes in the cortex (MOG and MBP) and the striatum (MBP) of BACHD rats at 12 weeks of age compared with WT littermates (*n* = 5–6/genotype; male). (**C**) Reduced cortical MBP protein levels in BACHD rats compared with WT littermates at 12 months of age (*n* = 3/genotype; male). (**D**) At 3.5 months of age, BACHD rats have higher average g-ratios, and the frequency distribution of the g-ratios of round healthy axons are shifted to the right, suggesting larger numbers of axons with thinner myelin sheaths. (B, C) All values were normalized to their respective WT littermate levels. * *P* < 0.05; *** *P* < 0.001.

To further validate the WM deficits we observed in the YAC128 mice, we evaluated select WM measures in the BACHD rats, a transgenic model expressing full-length human mutant HTT with 97 polyglutamine repeats (23). BACHD rats display a number of progressive behavioural and neuropathological phenotypes that mimic clinical and pathological features seen in patients with HD (23–25). Consistent with the observations in the YAC128 mice, BACHD rats exhibited significantly lower DT-MRI FA values in the anterior CC, the CG and the EC at 12 months of age compared with WT littermates, suggesting the presence of WM microstructural abnormalities (Fig. 4A). Similarly, the BACHD rats showed deficits in the levels of myelin-related genes at 12 weeks of age, with significantly reduced levels of MBP transcripts in the cortex (31%) and striatum (38%), and cortical levels of MOG (17%) transcripts (Fig. 4B). The transcriptional changes in MBP were paralleled on the protein level as shown by reduced cortical MBP protein at 12 weeks of age (Fig. 4C). In addition, the ultrastructural properties of the BACHD rats were consistent with the YAC128 mice, with increased average G-ratio and higher frequency distribution of larger G-ratios pointing to overall thinner myelin sheaths (Fig. 4D). Taken together, these findings indicate that WM-related microstructural and molecular changes are not unique to the YAC128 mice, but are a common early disease feature that reflects mutant HTT-induced pathology.

## Discussion

**Figure 5.**
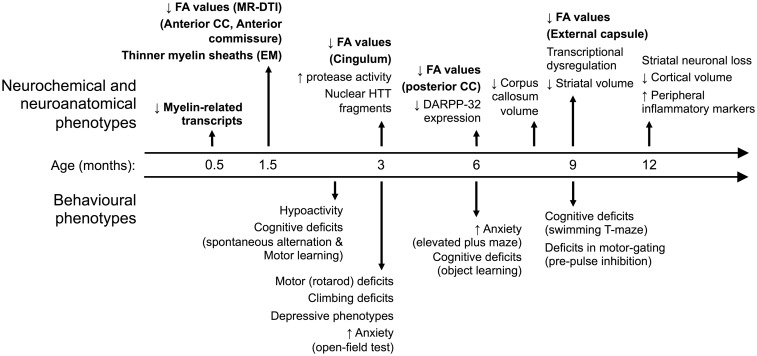
Time-course of behavioural, neurochemical and neuroanatomical phenotypes in the YAC128 HD mice. YAC128 HD mice manifest phenotypes that mimic several features of human HD including progressive motor and cognitive deficits, and depressive and anxiety-like behaviours. The YAC128 mice develop neuropathological changes reminiscent of HD including not only preferential striatal atrophy but also early myelination and white matter abnormalities. The YAC128 phenotypes identified in the present study are shown in bold.

WM abnormalities are a well-established pathological feature of HD, although their molecular and structural characteristics and aetiology have been largely unexplored. In this study, we demonstrate that abnormalities in WM microstructure manifest in YAC128 HD mice well before any neuronal loss. We show that WM abnormalities appear progressively in different WM-rich regions, starting with the anterior CC and AC at 1.5 months, the CG at 3 months, the posterior CC at 6 months and the EC at 9 months of age. We further show that the abnormalities in WM microstructure are paralleled by the presence of thinner myelin sheaths and lower levels of myelin-related gene transcripts in these mice. We also show that the WM microstructural abnormalities and lower myelin-related gene transcripts are present in a different rodent model of HD, the BACHD rats. These findings (summarized in Fig. 5) suggest that WM pathology in HD is an early event and may be, at least in the nascent stages of the disease process, independent of neuronal loss.

Our findings support previous evidence of WM abnormalities in animal models of HD. Assessment of brain structural changes revealed progressive atrophy of the myelin-rich CC region in brains of YAC128 HD mice (26). Furthermore, the volume of the CC was the most discriminatory structure for discerning YAC128 from WT mice, indicating the occurrence of high levels of atrophy specifically in this region of the brain in HD (26). Diffusion MR imaging studies have also shown abnormal WM microstructure in the R6/2 and HdhQ250 mouse models, and in a transgenic rat model of HD carrying a truncated *HTT* fragment with 51 CAG repeats (27–29). Thinner myelin sheaths, reflected by higher g-ratios, have been reported for the BACHD and HdhQ250 mouse models (27,29). Similar to our findings, these alterations in myelin sheaths were paralleled by reduced expression of myelin-related genes, such as MBP and MOG, in R6/2 and HdhQ250 HD mice (27,29).

We show that the deficits in myelin-related gene transcripts can be seen in differentiated primary oligodendrocytes isolated from YAC128 HD mice, suggesting that the deficits are a consequence of cell-intrinsic effects of mutant HTT in oligodendrocytes. A number of potential pathogenic mechanisms consistent with a cell-intrinsic origin of abnormal myelination in HD have been raised previously. The peroxisome-proliferator-activated receptor gamma coactivator 1 alpha (PGC1alpha), a transcriptional regulator of energy metabolism, has been suggested to play a role (27,30). PGC1alpha activity is compromised in a number of models of HD (31–34). Impaired PGC1alpha activity, as a result of PGC1alpha knockdown or overexpression of mutant HTT, has been shown to reduce the levels of MBP and a number of genes involved in the biosynthesis of cholesterol, a major constituent of myelin, in oligodendrocytes (27). This is in agreement with previous studies demonstrating defects in cholesterol biosynthesis in a number of animal models of HD, including the YAC128 HD mice (35,36). The myelin regulatory factor (MYRF), a master transcriptional regulator of myelin-related gene expression in mature oligodendrocytes, has also been implicated in HD, and reduced expression levels of MYRF were observed in the HdhQ250 mouse model (29). Furthermore, mutant HTT was found to interact with MYRF and affect its transcriptional activity, leading to reduced expression of myelin-related genes (37). It should be noted that while these observations support direct effects of mutant HTT on oligodendrocytes, we cannot exclude the contribution of additional non-cell intrinsic effects *in vivo*.

In addition to myelinating axons to insulate them electrically and facilitate saltatory conduction along their tracts, oligodendrocytes play important roles in maintaining axonal integrity and function (38–40). Oligodendrocytes secrete growth factors, including BDNF, IGF-1 and GDNF, that have neuroprotective effects on neurons (38,40). Oligodendrocytes also provide axonal metabolic support through mechanisms that include export of lactate through monocarboxylate transporters (38,41,42). The importance of the latter was demonstrated in a recent study showing that compromised function of the oligodendroglia-enriched monocarboxylate transporter 1 results in axonal damage and neuronal degeneration (43). Thus, it is interesting to speculate whether, in addition to the deficits in myelination we describe, mutant HTT interferes with other oligodendroglial functions which are important to axonal integrity, and whether such impairments contribute to axonal degeneration and neuronal dysfunction in HD (44).

An important consideration is whether oligodendrocyte dysfunction and associated abnormalities in myelination and WM structure contribute to the clinical manifestations of HD. Indeed, proper myelination is integral for rapid propagation of action potentials, and loss of myelination is associated with severe disabilities in a number of brain disorders (45). In HD, a number of studies have found significant correlations between indices of WM structural integrity such as MR-DTI FA and neuropsychiatric symptoms, including depression, irritability and apathy (46–48). Furthermore, distinct patterns of abnormalities in discrete WM tracts are correlated with motor and cognitive deficits in HD (49). In mice, expression of mutant HTT specifically in mature oligodendrocytes under the proteolipoprotein promoter was sufficient to induce a number of pathological and behavioural abnormalities, including progressive body weight loss, impaired motor performance and reduced survival (37). These findings indicate that WM abnormalities are associated with clinical outcomes and functional decline, and may indeed contribute to the pathological and behavioural manifestation of HD. This raises the possibility that interventions aimed at restoring oligodendrocyte function and proper myelination may confer therapeutic benefits in HD.

There have been no major efforts aimed specifically at ameliorating oligodendrocyte dysfunction and maintaining WM integrity in HD, but some evidence of potential therapeutic value exists. Improved callosal WM microstructure as a result of rhythm exercise was associated with improved executive function in HD (50). Immunotherapy-mediated inhibition of semaphorin 4D, a negative regulator of oligodendrocyte precursor cell migration and differentiation, prevented atrophy of the CC and improved behavioural phenotypes in YAC128 HD mice (51). These studies suggest that WM-targeted therapeutic approaches may be beneficial in HD, though this is yet to be directly tested.

In conclusion, we demonstrate that WM abnormalities occur early, prior to any neuronal loss, and are paralleled by myelination deficits and reduced myelin-related gene expression by oligodendrocytes in YAC128 HD mice and BACHD rats. Our findings support the need for future studies to address the contribution of WM abnormalities to the clinical manifestations of HD and to evaluate their potential as targets for therapy in HD.

## Materials and Methods

### Animals

For the YAC128 experiments, male and female mice (line 53) expressing a full-length human HTT transgene with 128 CAG repeats maintained on the FVB/N strain (8) were housed with littermates of mixed genotype in groups of 2–5 on a 12 h light/dark cycle with free access to food and water. All experiments were performed with the approval of the Institutional Animal Care and Use Committee at Biological Resource Centre (BRC), A*STAR. For the BACHD rats, experiments were approved by the local ethics committee at Regierungspraesidium Tuebingen, and carried out in accordance with the German Animal Welfare Act and the guidelines of the Federation of European Laboratory Animal Science Associations, based on European Union legislation (Directive 2010/63/EU).

### Real-time quantitative PCR

YAC128 mice: Microdissected striatal and cortical tissues of YAC128 HD and WT littermate controls (9 per genotype, all males) were snap frozen, and total RNA was extracted using the RNeasy Mini Kit (Qiagen). For primary culture experiments, total RNA from OPCs was extracted from a minimum of two pooled pup cortices per independent biological replicate (seven pooled biological replicates per genotype, mixed sex) using the PureLink RNA Micro Kit (Invitrogen). First-strand cDNA synthesis was performed using the SuperScript First Strand Synthesis System (Invitrogen). Following preliminary runs with selected primers from Primerbank (http://pga.mgh.harvard.edu/primerbank/, last accessed April 28, 2016) on WT cortical cDNA, the mouse-specific primers listed in Table 1 were subsequently used on all samples. All probes were run in triplicate with Sybr Select Universal Master Mix (Invitrogen) on a StepOnePlus (ABI) to obtain Ct values.

BACHD rats: Rat brains were rapidly dissected on ice and stored at −80 °C. Total mRNA was extracted from frozen tissues of the striatum and cortex using the RNeasy Lipid Tissue middle/Large Kit and cDNA was synthesized using a QuantiTect Reverse Transcription Kit following the manufacturer’s instructions (Qiagen, Germany). Real-time PCR using QuantiTect SYBR Green PCR Kits (Qiagen, Germany) was performed for the analyses of the expressions of MOG, MBP and CNP at cDNA level in both striatum and cortex. Absolute quantification of cDNA was performed using the Light Cycler 480 instrument with the aid of built-in Light Cycler software. ATP5B and Eif4a were chosen as reference genes for analyzing the cDNA levels in the striatum and ATP5B and CanX3 for the analyses in the cortex. Forward and reverse primers (Table 1) of a gene were designed to be at least one intron apart and to cover all transcription variants of the target gene. Data were compared between male BACHD TG5 rats and WT littermates at 3 months of age (*n* = 6). One transgenic rat and one WT rat were excluded for the analysis of MBP expression due to the extremely low CT values of both housekeeping genes detected by real-time PCR.

### *Transmission* EM

Animals (3 per genotype, all males) were transcardially perfused with 2.5% glutaraldehyde and 2.5% PFA in 0.1 m sodium cacodylate buffer before post-fixing the brains overnight at 4**°**C in the same buffer, and then subsequently washing in phosphate buffered saline. Brain samples were sent to the Harvard Medical School EM unit for further processing. Briefly, coronal slices at the level of Bregma -1 mm were made from the central part of the CC before post-fixation in 1% osmium tetroxide/1.5% potassium ferrocyanide solution for 1 h. After washing with 1% uranyl acetate in maleate buffer, the samples were dehydrated, infiltrated with epon, embedded and polymerized at 60**°**C for 2 days. Ultra-thin slices (100 nm) were cut before imaging on a transmission electron microscope. For image analyses, Stereoinvestigator v11 was employed to allow for systematic random selection of axons, with a minimum of 500 axons analysed per brain.

### MRI acquisition and analysis

YAC128 HD mice: 16 mice (8 WT; and 8 YAC128; 50% male) were scanned at 1.5, 3, 6, 9 and 12 months old. The mice were anaesthetized using 1–3% isoflurane mixed with oxygen and air (1:1 ratio) through a nose cone during preparation and MRI scanning. Respiration rate was maintained at 80 ± 10 bpm and temperature at 36–37°C with an MRI-compatible heater (SAII, NY). MRI was conducted on a 7T scanner (ClinScan, Bruker BioSpin, Germany) with four-channel mouse brain array coils. DTI was acquired using a spin-echo EPI sequence with eight averages of 30 diffusion sensitizing directions, b = 1500 s/mm^2^, repetition time (TR) = 10000 ms, echo time (TE) = 40 ms and 0.2 × 0.2 × 0.5 mm^3^ voxel resolution. The acquisition time was 45.5 min. High-resolution structural MRI was acquired with fast spin-echo with TR = 2760 ms, TE = 43 ms and voxel size = 0.1 × 0.1 × 0.3 mm^3^. After eddy current distortion and motion correction, tensor fitting was carried out using the weighted least squares method with FDT (FMRIB's Diffusion Toolbox, FSL, Oxford; http://fsl.fmrib.ox.ac.uk/fsl/, last accessed April 28, 2016) to obtain FA. For each time point, one animal with the highest signal-to-noise ratio was chosen to be the initial target, and the remaining animals' FA images were non-linearly registered to it using FNIRT (FMRIB’s nonlinear image registration tool). The registered images were then averaged to create time point-specific FA templates. Another round of non-linear registration was carried out to align all FA images to the corresponding FA template. After in-plane Gaussian smoothing of 0.3 mm full-width at half maximum, voxel-wise group analysis was conducted on the FA map using two-sample t-tests in SPM8 (http://www.fil.ion.ucl.ac.uk/spm/, last accessed April 28, 2016). The statistical analysis was restricted to WM regions with FA > 0.175 on the template. Family wise correction was performed with a cluster threshold of *P* < 0.05 determined by a Monte-Carlo simulation using 3DClustSim in AFNI (NIH; http://afni.nimh.nih.gov/, last accessed April 28, 2016). Based on the results of the voxel-wise analysis, regions of interest were manually drawn in the anterior CC, posterior CC, AC, EC and CG on the templates (Fig. 1B). The average FA values in those regions were calculated.

BACHD rats: Diffusion tensor images were acquired using an echo planar imaging with 256 directions (b = 0,1000s/mm^2^, 54×21 mm field of view, 128×52 mm matrix, twenty-six 1 mm slices, 60/5500ms TE/TR). FA images were generated using Inveon Acquisition Workplace. Inveon Research Workplace software was used for the image analysis, where a volume of interest approach was implemented on anterior and posterior CC, the CG and the EC. FA values were compared between male BACHD rats and WT controls at 12 months of age (TG5:WT = 14:13 for EC, TG5:WT = 11:10 for the remaining areas).

### Primary culture and differentiation of OPCs

Cortices from P6-P7 pups (seven pooled biological replicates per genotype, mixed sex) were dissected and subsequently dissociated with the Neural Tissue Dissociation Kit from Miltenyi. Anti-O4 magnetic microbeads (Miltenyi) were then used to isolate a pure population of O4+ OPCs. For culture and differentiation, serum- and antibiotic-free medium comprising 2% B27 (Gibco) and 30 ng/ml thyroid hormone (Sigma) in Neural Basal Media (Gibco) was used. Medium was changed daily and cells were harvested after 7 days.

### Western blot

To access protein expression level of MBP in BACHD rats, western blot was applied using striatal brain lysis of male BACHD TG5 rats and WT controls at 3 months of age (*n *= 3). The striata were stored at −80 °C after dissection and were homogenized with a tissue homogenizer at a speed of 30 000 rpm for 30 s in 10 volumes (w/v) modified RIPA buffer (50 mm Tris pH 7.5, 150 mm NaCl, 1% NP40, 0.5% deoxycholate, 0.1% SDS) with Complete Protease Inhibitor Cocktail tablets (Roche, Germany). After a further 5-min sonication with bath sonicator for shearing genomic DNA, the lysates were centrifuged at 4 °C for 15 min at 16 200*g*, and the supernatant was used for western blot analysis (52). The blot was probed with polyclonal rat antibody recognizing rat MBP (1: 5000, ab40390, abcam, USA) followed by incubation in the HRP-conjugated secondary antibody (1:10 000, ab191866, abcam, Cambridge, MA, USA). Finally, blots were developed with ECL Western Blotting Detection Reagent (RPN2134, Amersham Biosciences, Germany) and followed by visualization on X-ray film (AGFA, Germany).

### Statistical analysis

Graphpad Prism v6 was used for statistical analyses and data are expressed as means ± SEM. Two-way ANOVA was performed to assess the effects of genotype and age on FA. Unless otherwise stated, pair-wise comparisons between genotypes at individual time points were assessed with a Student’s t-test. Differences were considered statistically significant when *P* < 0.05.

## Supplementary Material

Supplementary Material is available at *HMG* online.

Supplementary Data

Supplementary Data
