# Supplementary material for: Structural and molecular myelination deficits occur prior to neuronal loss in the YAC128 and BACHD models of Huntington disease
Source: Hum Mol Genet. 2016 Apr 28;25(13):2621–32. doi: 10.1093/hmg/ddw122 (PMC5181633; doi:10.1093/hmg/ddw122)
Supplement: Supplementary Data [file supp_25_13_2621__index.html]

Structural and molecular myelination deficits occur prior to neuronal loss in the YAC128 and BACHD models of Huntington disease — Supplementary Data 

# Structural and molecular myelination deficits occur prior to neuronal loss in the YAC128 and BACHD models of Huntington disease

## Supplementary Data

files

- Supplementary Data - pdf file
