# Supplementary material for: Structural and molecular myelination deficits occur prior to neuronal loss in the YAC128 and BACHD models of Huntington disease
Source: Hum Mol Genet. 2016 Apr 28;25(13):2621–32. doi: 10.1093/hmg/ddw122 (PMC5181633; doi:10.1093/hmg/ddw122)
Supplement: Supplementary Data [file supp_ddw122_Teo_et_al_Supplementary_data.pdf]

Supplementary data:

Structural and molecular myelination deficits occur prior to neuronal loss in the YAC128 and BACHD models of Huntington disease

Supplementary Table 1. Two-way ANOVA of mean FA in regions of interest (related to Figure 1)

		Anterior CC		Posterior CC		CG		AC		EC	
Source of Variation	*degree of freedom*	*F*	*P-value*	*F*	*P-value*	*F*	*P-value*	*F*	*P-value*	*F*	*P-value*
Genotype	1	**24.97**	< 0.0001	**14.48**	0.0003	**18.45**	0.0001	**23.02**	< 0.0001	**4.22**	0.04
Age	4	**3.02**	0.02	1.94	0.11	1.14	0.34	**5.95**	0.0004	2.37	0.06
Interaction	4	0.92	0.46	0.47	0.76	0.75	0.56	0.63	0.64	0.91	0.47

FA – fractional anisotropy; Dr – radial diffusivity; Dp – parallel diffusivity; AC – anterior commissure; CC – corpus callosum; CG – cingulum; EC – external capsule. Significant results highlighted in bold.
